# Dummy regression to predict dry fiber in Agave lechuguilla Torr. in two large-scale bioclimatic regions in Mexico

**DOI:** 10.1371/journal.pone.0274641

**Published:** 2022-09-15

**Authors:** José Óscar M. López-Díaz, Jorge Méndez-González, Pablito M. López-Serrano, Félix de J. Sánchez-Pérez, Fátima M. Méndez-Encina, Rocío Mendieta-Oviedo, Librado Sosa-Díaz, Andrés Flores, Emily García-Montiel, Víctor H. Cambrón-Sandoval, Alejandro Zermeño-González, José J. Corral Rivas

**Affiliations:** 1 Consultor Forestal Independiente, Saltillo, Coahuila, México; 2 Departamento Forestal, División de Agronomía, Universidad Autónoma Agraria Antonio Narro, Saltillo, Coahuila, México; 3 Instituto de Silvicultura e Industria de la Madera, Facultad de Ciencias Forestales y Ambientales, Universidad Juárez del Estado de Durango, Durango, México; 4 Consultor Estadístico Independiente, Saltillo, Coahuila, México; 5 Estudiante de Postgrado en Ciencias Forestales, Colegio de Postgraduados, Montecillo, Texcoco, Estado de México, México; 6 Cenid-Comef, Instituto Nacional de Investigaciones Forestales, Agrícolas y Pecuarias, Alcaldía Coyoacán, México; 7 Facultad de Ciencias Forestales y Ambientales, Universidad Juárez del Estado de Durango, Durango, México; 8 Facultad de Ciencias Naturales, Universidad Autónoma de Querétaro, Querétaro, México; 9 Departamento de Riego y Drenaje, División de Ingeniería, Universidad Autónoma Agraria Antonio Narro, Saltillo, Coahuila, México; National Institute of Technology Silchar, India, INDIA

## Abstract

*Agave lechuguilla* Torr., of the family Agavaceae, is distributed from southwestern United States to southern Mexico and is one of the most representative species of arid and semiarid regions. Its fiber is extracted for multiple purposes. The objective of this study was to generate a robust model to predict dry fiber yield (*Dfw*) rapidly, simply, and inexpensively. We used a power model in its linear form and bioclimatic areas as dummy variables. Training, generation (80%) and validation (20%) of the model was performed using machine learning with the package ‘*caret’* of R. Using canonical correlation analysis (CCA), we evaluated the relationship of *Dwf* to bioclimatic variables. The principal components analysis (PCA) generated two bioclimatic zones, each with different *A*. *lechuguilla* productivities. We evaluated 499 individuals in four states of Mexico. The crown diameter (*Cd*) of this species adequately predicts its fiber dry weight (R^2^ = 0.6327; p < 0.05). The intercept *(β*_0_), slope [*lnCd (β*_1_)], zone [(*β*_2_)] and interaction [*lnCd*:Zona (*β*_3_)] of the dummy model was statistically significant (p < 0.05), giving origin to an equation for each bioclimatic zone. The CCA indicates a positive correlation between minimum temperature of the coldest month (Bio 6) and *Dwf* (r = 0.84 and p < 0.05). In conclusion, because of the decrease in Bio 6 of more than 0.5°C by 2050, the species could be vulnerable to climate change, and *A*. *lechuguilla* fiber production could be affected gradually in the coming years.

## 1. Introduction

In Mexico, arid and semiarid regions cover 54% of the territory and are inhabited by approximately 40% of the country’s population [1]. Their vegetation is scrub Crassicaule, Desert Microphyll, and Desert Rosetophile; the genera Larrea, Agave, Dasylirion, Yuca, etc., are the most representative [2]. One of the most economically and ecologically important species in arid regions is *Agave lechuguilla* Torr., a succulent plant of the Agavaceae family, distributed from southeastern United States to southern Mexico over 11 federal states [3, 4], covering 142 115 km^2^ [5].

This species, known as “lechuguilla”, is outstanding for its fiber (“ixtle”), which is extracted from its bud (”cogollo”) once the plant has reached 25 cm in height [6]. The fiber is used in the automobile industry, cordage, rugs, and cleaning brushes, among others [7]. Lechuguilla fiber can be extracted manually or mechanically [3, 8]. The former method produces better quality and can sell for a price of up to 1.2 US dollars per kg. With this technique, 1.87 kg h^-1^ is produced, while the mechanical method can produce between 15 and 18 kg h^-1^ of lower quality fiber [9]. Better quality fiber is obtained from bud (“cogollos”) with young leaves, which contain less lignin than leaves from the plant crown [8]. On lechuguilla plantations, the bud takes seven to eight months to regenerate (to reach ≥ 25 cm of height), while naturally it takes between 16 and 24 months [10].

The fiber from *A*. *lechuguilla* is crucial for inhabitants of arid and semiarid regions of the country [11], benefitting nearly 52,000 families [7]. For this reason, it is necessary to quantify fiber existences. This can be done directly by harvesting the plant or indirectly using allometric models. In Mexico there are only three studies on estimating *A*. *lechuguilla* fiber dry weight: Blando & Baca [12], Pando *et al*. [13] and Velasco *et al*. [3]. All these studies were conducted at a local scale, and some of these models require harvesting the entire plant or part of it [8, 14] to estimate fiber weight. Since the objective of creating a model is to make predictions with new data, it is necessary that the model comply with all the statistical assumptions, and it is especially important to evaluate its predictive capacity [15].

For this reason, it is crucial to generate robust allometric models to estimate *A*. *lechuguilla* fiber dry weight with non-destructive techniques that are simple, rapid, and efficient, and that solve the problem of scale, considering the productivity areas and determining the relation of fiber dry weight to bioclimatic variables. The objective of this study was to create a model of this type that will serve not only to support government procedures [16] for authorizing programs for use of the fiber but also for decision-making in management and conservation of the species.

## 2. Materials and methods

### 2.1. Description of the study area

The study was conducted in the arid and semiarid regions in northeastern Mexico in the states of Chihuahua (Chih), Coahuila (Coah), San Luis Potosí (SLP) and Zacatecas (Zac). *Agave lechuguilla* grows in colluvial, sandy loam, limestone and clay soils [7, 17] in areas where precipitation varies from 150 to 500 mm, at altitudes from 200 to 2400 m [2, 11] and temperatures of 3 to 30°C [18], and even in extreme temperatures of -8 to 44°C. It is known that this plant can withstand droughts and floods [19].

### 2.2. Bioclimatic zoning of distribution areas of *Agave lechuguilla* Torr.

We obtained the following bioclimatic variables from the website https://chelsa-climate.org/: mean annual temperature, Bio 1 (°C), maximum temperature of the hottest month, Bio 5 (°C), minimum temperature of the coldest month, Bio 6 (°C), and mean annual precipitation, Bio 12 (mm) in raster format at a resolution of 1 km^2^ [20]. Using ArcMap 10.4.1, 1000 points were distributed randomly over the area of *A*. *lechuguilla* distribution in the country, for which the values of each bioclimatic variable and of altitude (m) obtained from the Modelo Digital de Elevación Mexicano (https://www.inegi.org.mx/app/geo2/elevacionesmex/) were extracted. With this information, a principal components analysis (PCA) was performed in R software [21] using the library ‘FactoShiny’ [22] which requires ‘FactoMiner’ [23], using the variables in standardized form to extract the first three principal components. Then, with the unrotated eigenvalues derived from PC1, an interpolation (Inverse Distance Weighted) was carried out in ArcMap, from which isolines spaced at 2.85 were derived.

### 2.3. Sampling *Agave lechuguilla* Torr for fiber extraction

*Agave lechuguilla* sampling was selective only in “ejidos” with gathering permits. therefore, a special permit was not necessary to harvest the individuals in this study.

Plants ≥ 10 cm tall were collected (according to the gatherers, it is not until the plant reaches this height that the bud can be worked traditionally). Using a 3 m tape measure, we measured average crown diameter (*Cd*, cm) and total height (*H*, cm) on each selected plant. Prior to felling the plant (to obtain aerial biomass), the bud was extracted using a “cogollera” (a rustic instrument characteristic of the gatherers) to measure its length (*Lc*) and immediately extract the fiber manually. The fresh fiber was placed in a paper bag properly labeled and taken to the laboratory at the UAAAN to be dried in a Thermo Scientific^TM^ HERAthem^TM^ (Modelo OMH750) oven at 70°C until it had a constant weight, which was obtained with a Torrey® (Modelo L-EQ) scale with a capacity of 5 kg and precision of 1 g.

### 2.4. Generation and validation of the equation for estimating dry fiber weight

To predict dry fiber (*Dfw*) of individual *A*. *lechuguilla* plants, the allometric power equation (Eq 1) was tested in its linear form (Eq 2), applying logarithms to correct error variance [15], as used by Wood [24], Zárate *et al*. [25] and Flores et al. [26].

$$Y=\beta_{0}X^{\beta_{1}}\varepsilon$$ (1)

$$\ln\left(Y\right)=ln\beta_{0}+\beta_{1}\times \ln(X)+\varepsilon$$ (2)

where *Y* = fiber dry weight (kg), testing crown diameter (*Cd*, cm), total plant height (*H*, cm), and the product of the two, *Cd*×*H* (cm×*cm*), as predictor variables, *ln* = natural logarithm and *β*_0_ and *β*_1_ = coefficients of regression.

Eq 2 was fit using the bioclimatic zone as the dummy variable $D=\left\{ \begin{array}{c} 0\;(Zone\;1)\\ 1\;(Zone\;2) \end{array}\right.$ considering an effect to the intercept (*Zone*) and the model slope (*X*: *Zone*) to determine the existence of a model (for the entire area) or one for each bioclimatic zone, as follows:

$$\ln\left(Y\right)={ln\beta }_{0}+\beta_{1}\times (lnX)+\beta_{2}\times (Zone)+\beta_{3}\times (lnX:Zone)+\varepsilon$$ (3)

With 80% of all the data (obtained randomly by quantiles) and using the method of ordinary least squares [15], the model was trained using machine learning (ML) with the package R ‘caret’ [27]. With the same library, the model was then generated by bootstrap (*n* = 25). Hypothesis tests (∝ = 0.05) were performed on the coefficients of regression ($H_{0}:\;\beta_{i}=0\;vs\;H_{1}:\beta_{i}\neq 0,\ldots ,\beta_{ij}$) of the final model. Statistical tests ((∝ = 0.05) were applied to assure compliance to assumptions of normality [Lilliefors (Kolmogorov-Smirnov)], variance homogeneity (Breusch-Pagan), and independence of the errors (Durbin-Watson). Possible atypical data (−3≤*r*_*i*_≥+3) were examined through studentized residuals *r*_*i*_. Influence on the coefficients of the model, a) Cook distance, *D*_*i*_ (observation *y*_*i*_), and on the precision of COVRATIO estimations [15, 28, 29], and b) DFBETAS (${\hat{\beta }}_{i}$). DFFITS (influence of the *i*^th^ observation on the predicted values), and on the precision of the estimations. The total of the basic hypotheses of the regression model is summarized in the following expression: *ε*_*i*_~*N*(0, *σ*^2^), *i* = 1,…*n*; that is, random, independent, and identically distributed errors, according to a normal distribution with mean zero and variance *σ*^2^.

After verifying compliance to all the previous assumptions, validation of the final model was carried out in ‘caret’ [27] with 20% of the independent data, using four methods: a) Leave One Out Cross-Validation, b) k-fold validation (k = 10), c) Repeated K-fold (k = 10 & 3 sets) and d) Bootstrap Cross-Validation, calculating the Coefficient of Determination (R^2^), Root Mean Square Error (RMSE) and Mean Absolute Error (MAE).

The return of *ln* to its original units to predict *A*. *lechuguilla* fiber dry weight of the resulting equation is not direct since, if the distribution of a *log Y* is normal, a *log X*, result of the antilogarithm *log Y*, may be not normal and biased since the median is obtained and not the mean [24]. This would have to be corrected by applying to the final model a correction factor (calculated here), which is given by: $CF=e^{\left({SEE}^{2}/2\right)}$, where SEE is the estimation error of the regression [30].

### 2.5. Canonical correlation analysis

With the aim of identifying the dependence of *A*. *lechuguilla* dry fiber with environmental variables, as well as the interdependence between factors (matrices *X*, *Y*): [*Cd*, *H* and *Dfw*], [Bio 1, Bio 5, Bio 6, Bio 12 and altitude], a Canonical Correlation Analysis was performed using the package R ‘CCA’ [31] of R statistical software [21]. The matrices *X*, *Y* were generated with the average of the variable per municipality (S1 Table).

## 3. Results and discussion

### 3.1. Bioclimatic zoning

**Fig 1:**
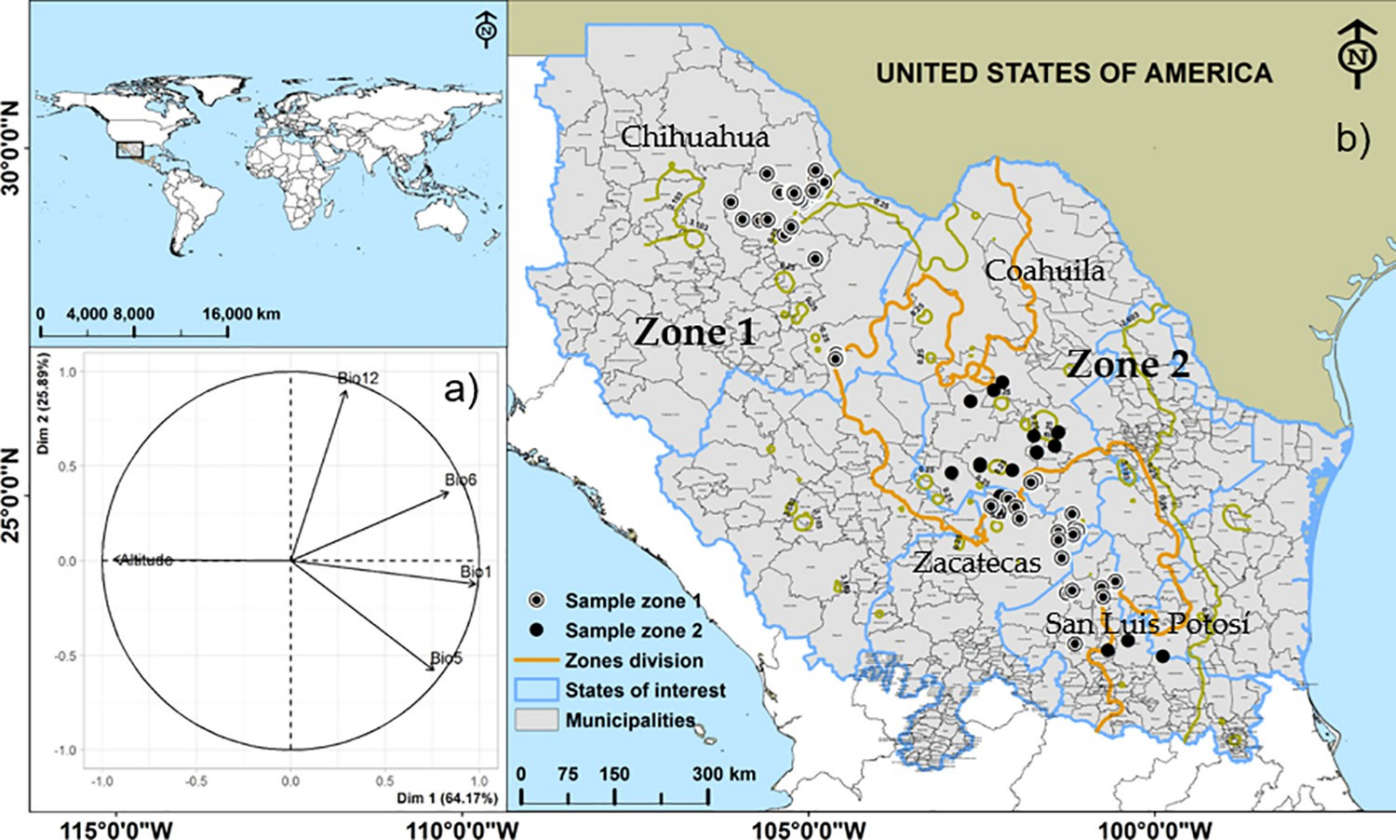
Location of the study area in the global context (a), two bioclimatic zones generated of *Agave lechuguilla* Torr. using principal components analysis (PCA) without rotation, where brown lines indicate the limits of the bioclimatic zones; circles indicate the sampling sites of the species; while the polygons (blue lines) indicate the states of the country, and thin black lines indicate municipal boundaries (b). Correlation plot of bioclimatic variables and altitude with eigenvectors of components 1 and 2 of the principal components analysis (c). [Shapefiles were taken from: world map [34], State Political Division of Mexico [35] and Municipal Political Division of Mexico [36]. Fig 1A may be similar, but not identical to any published figure, however, in this paper it is used solely for illustrative purposes, no information or databases were extracted from it.

The results of the PCA showed that components 1 (64.17%) and 2 (25.89%) explain 90.07% of the variance. The contribution of each variable in component 1 was 29.98% (Bio 1), 27.61% (altitude), 21.80% (Bio 6), 17.95% (Bio 5), 2.66% (Bio 12) (Fig 1A). The unrotated scores (eigenvalues = 0.25) gave origin to two bioclimatic zones divided almost longitudinally: Zone 1: Chih, Zac, and the northern part of SLP (n = 200); Zone 2: Coah, southeastern SLP and northeastern Zac (n = 199) (Fig 1B).

Using the same variables, plus slope, aspect and records of the presence of the species, some authors [32, 33] have generated zones of productive potential in this region of the country, estimating between 9 and 5 million ha that are ideal for *A*. *lechuguilla* plantations, mainly in zone 2 of our study (Fig 1B). Flores *et al*. [26] constructed models to estimate aboveground biomass of this species, considering the state boundaries (Fig 1) as the dummy variable (or zone) and found differences in biomass among the zones.

**Fig 2:**
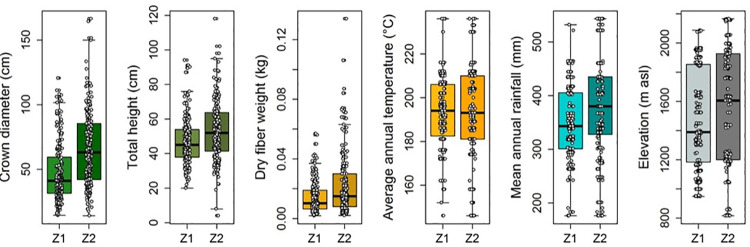
Boxplot with Jitter of dendrometric (first three plots) variables of *Agave lechuguilla* Torr. and values of bioclimatic variables obtained from each sampling site of individuals of this species (last three plots). Where Z1 = Zone 1 and Z2 = Zone 2, represents the bioclimatic zones generated through principal components analysis (Fig 1).

When we examined the means of the dendrometric (*Cd*, *H* and *Dfw*) and bioclimatic (Bio 1, Bio 5, Bio 6, Bio 12 and altitude) variables through dummy-regression models equivalent to Student “t” tests) in logarithmic form for compliance to variance normality and homogeneity: *ln*(*Y*) = *β*_0_+*β*_1_×*Zone*, we found that there were statistically significant differences among zones (p ≤ 0.05). In Zone 2, *A*. *lechuguilla* height, crown diameter, and quantity of fiber were higher than in Zone 1. This appears to obey basically minimum temperature (Bio 6) and, to a lesser degree, precipitation (Bio 12), which are also the highest (Fig 2, S2 Table) in this zone. There was no proof for rejecting the *H*_0_ that mean annual precipitation and altitude are the same among zones (p = 0.956 and p = 0.106).

### 3.2. Characterization of the *Agave lechuguilla* Torr. sample

The total number of plants evaluated was 499, distributed geographically over a distance of more than 1000 km, according to Fig 1: (Chih = 146, Coah = 119, SLP = 124 and Zac = 110). The plants were obtained from 20 municipalities and 46 ejidos (S1 Appendix 1). It can be seen that *A*. *lechuguilla* individuals are taller (52.76 cm) and crown diameters are larger (66.10 cm) in Zone 2 (Table 1, Fig 1), and therefore, they accumulate on average a larger amount of fiber in their plant tissues (22.21 g plant^-1^), but up to maximums of 34.15 and 46.60 g plant^-1^, according to percentile 95 (Zone 1 and Zone 2). In general, to predict *A*. *lechuguilla* dry fiber, a selective sampling is applied, considering a lower limit for plant height; for example, in Zone 2 of this study, Blando and Baca [12] selected plants ≥ 45 cm tall finding that the accumulation of dry fiber can be between 45.6 and 70.5 g plant^-1^, much higher than the average of our study. We included plants ≥ 10 cm tall, and for this reason, when averages (or even medians) are compared with previous studies, the differences are contrasting.

**Table 1:** Descriptive statistics of the sample used to predict dry fiber in *Agave lechuguilla* Torr.

Zone	Var	Mean	MeanCI’	0.05	0.10	0.25	Median	0.75	0.90	0.95	Range	SD	VCoef	MAD	IQR
**Zone 1 (n = 200)**	*Cd*	46.82	±3.20	19.98	23.00	31.50	41.00	59.25	83.60	94.53	106.00	22.93	0.49	17.42	27.75
	*H*	47.03	±1.88	28.95	32.00	38.00	45.00	54.00	62.83	71.10	74.00	13.49	0.29	11.86	16.00
	*Dfw*	14.19	±1.49	3.90	5.00	6.45	10.20	19.00	29.73	34.15	55.20	10.66	0.75	7.12	12.55
**Zone 2 (n = 199)**	*Cd*	66.10	±4.05	28.45	32.90	42.00	63.00	85.43	102.10	114.70	152.50	28.99	0.44	31.88	43.43
	*H*	52.76	±2.42	29.09	33.00	41.40	52.00	63.70	74.04	83.00	114.00	17.28	0.33	16.31	22.30
	*Dfw*	22.21	±2.87	4.00	5.00	8.00	15.00	30.00	0.05	46.6	132.00	20.48	0.92	13.34	22

Cd = crown diameter (cm), H = total height (cm), Dfw = dry fiber weight (g), MeanIC = confidence interval for the mean, 0.05, …, 0.95 = quantiles of the variable, SD = standard deviation, VCoef = coefficient of variation, MAD = median absolute deviation, IQR = interquartile range.

### 3.3. Analysis of predictors of *Agave lechuguilla* Torr. dry fiber

In a preliminary analysis Eq 2 was adjusted with each predictor (*Cd*, *H*, *Cd*×*H*). Although all show the capacity to predict *A*. *lechuguilla* dry fiber (0.519 < R^2^ < 0.633 and p-value< 2e-16), only *Cd* passes all the assumptions of a linear model (Table 2). For this reason, *Cd* was selected as the predictor of *A*. *lechuguilla* dry fiber. It has been demonstrated that the best predictors in this species are *Cd* and *H*, *e*.*g*. Valencia *et al*. [37] used *Cd* (cm) and plant volume (m^3^) to predict plant fresh weight (kg), with correlations of 0.9272 and 0.9433, respectively. Flores *et al*. [26] show that *Cd* and *H* efficiently predict aboveground biomass, explaining 91.4%. With destructive methods, it has been proven that heart volume (cm^3^) can explain from 57.9% [7, 25] to 90.98% [11] of the heart fresh weight. The moisture index (MPa) and the index of photosynthetically active radiation (mmol m^2^·S^-1^) explain 97% and 25% of the biomass of this species [38].

**Table 2:** Statistics of fit and verification of assumptions of the power model in logarithmic form of predictors of dry *Agave lechuguilla* Torr. fiber weight.

Coeff.	Estimate	S.E.	t-value	Pr(>|t|)	R^2^	KS	BP	D-W
**Intercept *(β***_**0**_ **)**	-9.132	0.186	-49.20	< 2e-16 ***	0.577	0.233	0.223	0.088
***lnCd (β***_**1**_ **)**	1.226	0.047	26.03	< 2e-16 ***				
**Intercept *(β***_**0**_ **)**	-11.027	0.289	-38.09	< 2e-16 ***	0.519	2.2e-16	3.58e-16	0.004
***lnH (β***_**1**_ **)**	1.733	0.750	23.18	< 2e-16 ***				
**Intercept *(β***_**0**_ **)**	-10.721	0.219	-48.92	< 2e-16 ***	0.633	0.003	0.202	0.102
***lnCdH (β***_**1**_ **)**	0.821	0.028	29.26	< 2e-16 ***				

Coeff = coefficient, S.E. = standard error, R^2^ = coefficient of determination. Value of p of the normality test, KS (Kolmogorov-Smirnov), Variance homogeneity, BP (Breusch Pagan) and independence, D-W (Durbin Watson). *Cd* = crown diameter, *H* = total plant height, *ln* = natural logarithm, *β*_0_ and *β*_1_ = coefficients of regression. Signif. codes: 0

’***’ 0.001

’**’ 0.01

’*’ 0.05 ’.’ 0.1 ’ ’ 1.

In addition, statistically significant (p < 0.001) results were obtained with the hypothesis tests on the coefficients of regression with different sample sizes (Table 3): intercept (*β*_0_), slope [*lnCd* (*β*_1_)], dummy variable [*Zone* (*β*_2_)] and interaction [*lnCd*:*Zone* (*β*_3_)] of Eq 3. This demonstrates correct bioclimatic zoning of *A*. *lechuguilla* productivity with the PCA and shows that the predictor (*lnCd*) of dry fiber does not originate from a random model.

**Table 3:** *p* values for the hypothesis tests of intercept, slope and interactions with the dummy variable at different sample sizes, with the power model in logarithmic form to predict *Agave lechuguilla* Torr. dry fiber.

	Sample size (%)						Mean (coef.)	S.D. (coef.)
Coefficient	100	90	80	70	60	50		
**Intercept (*β***_**0**_ **)**	2e-16	2e-16	2e-16	2e-16	2e-16	2e-16	-8.745	0.119
***lnCd* (*β***_**1**_ **)**	2e-16	2e-16	2e-16	2e-16	2e-16	2e-16	1.139	0.031
***Zone* (*β***_**2**_ **)**	1.0e-4	6.2e-5	0.001	1.2e-4	6.6e-5	5.4e-5	-1.375	0.236
***lnCd*:*Zone* (*β***_**3**_ **)**	0.020	0.003	0.015	0.002	0.002	9.2e-4	0.314	0.062
**R**	0.771	0.764	0.781	0.778	0.757	0.782		

*Cd* = crown diameter, *ln* = natural logarithm, *β*_0_,…, *β*_3_ = coefficients of regression, *Zone* = dummy variable, Mean (coef.) = mean of regression coefficients, S.D. (coef.) = standard deviation of regression coefficients. R = Coefficient of multiple correlation.

### 3.4. Model for predicting *Agave lechuguilla* Torr. dry fiber

**Fig 3:**
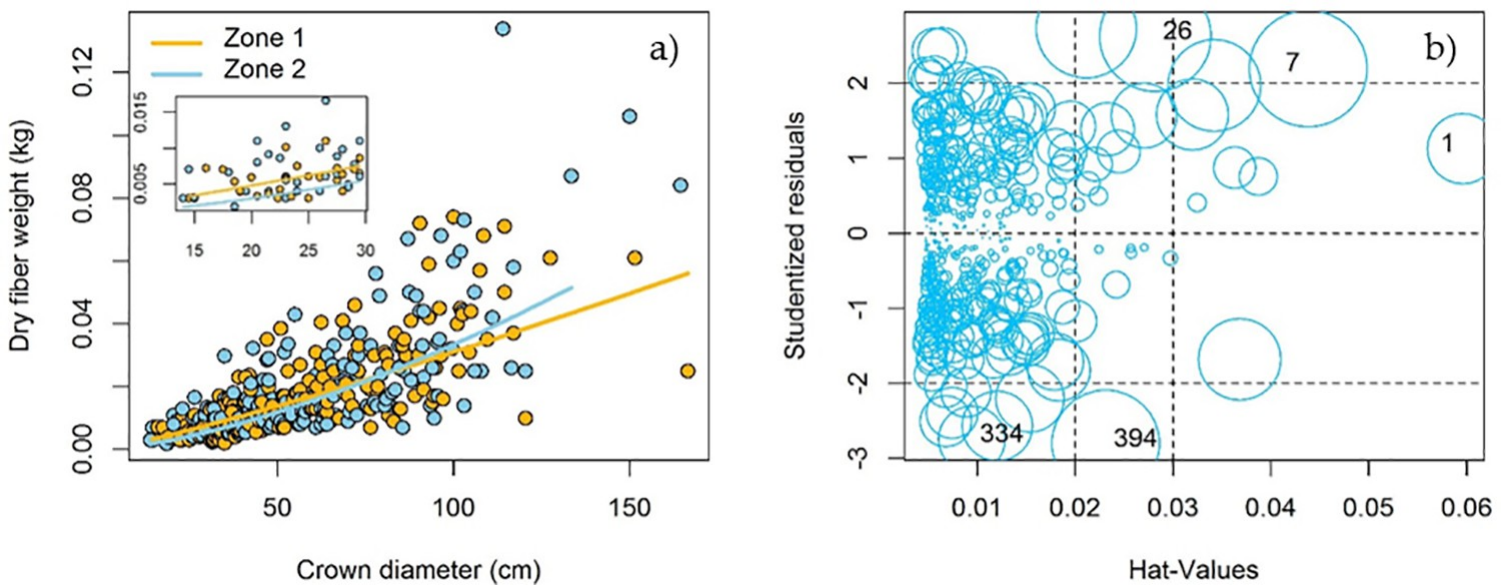
Observed data of dry fiber of *Agave lechuguilla* Torr. in bioclimatic zone 1 (brown circles) and bioclimatic zone 2 (blue circles) and data estimated with the dummy model in each bioclimatic zone (brown and blue lines, respectively) (a), analysis and representation of influential observations of the model (b).

The final model obtained with ML showed an R^2^ of 0.629. The coefficients of regression were highly significant, p<0.05 (Table 4), similar to the average obtained with different sample sizes (Table 3). The dummy variable (*Zone*) and the interaction *lnCd*:*Zone* show *p* values of 0.0004 and 0.0014 (Table 4), giving way to an equation for each bioclimatic zone (Fig 3A) for estimating *A*. *lechuguilla* dry fiber.

**Table 4:** Fit statistics of the power-dummy model in logarithmic form for estimating *Agave lechuguilla* Torr. fiber dry weight in arid and semiarid regions of Mexico.

Coefficients	Estimate	Std. error	t-value	Pr(>|t|)	I.C. 2.5%	I.C. 97.5%
**Intercept (*β***_**0**_ **)**	-8.826	0.252	-35.086	< 2e-16 ***	- 9.321	-8.332
***lnCd* (*β***_**1**_ **)**	1.162	0.066	17.640	< 2e-16 ***	1.033	1.292
**Zone (*β***_**2**_ **)**	-1.468	0.414	-3.546	0.0004 ***	- 2.282	-0.654
***lnCd*:Zone (*β***_**3**_ **)**	0.335	0.104	3.219	0.0014 **	0.130	0.540

I.C. = confidence interval, *Cd* = crown diameter, *ln* = natural logarithm, *β*_0_,…, *β*_3_ = coefficients of regression, *Zone* = dummy variable, Signif. codes: 0

’***’ 0.001 ’**’ 0.01 ’*’ 0.05 ’.’ 0.1 ’ ’ 1.

Because of the wide variation in fiber observed in this species (Table 1), in the regression analysis we present some influential observations (Fig 3B) on the model coefficients: *D*_*i*_ (DFFITS), but none with *D*_*i*_>1 that could be eliminated. There were no influential observations regarding DFBETAS. With COVRATIO, several observations were outstanding as influential, but we underline that no atypical *r*_*i*_ data were detected. The correction (1.127) and multiplicative factor obtained at the final equation can correct the bias and improve the estimations of *Dfw* by 12.7%.

In this way, the resulting equation for estimating *A*. *lechuguilla* fiber dry weight in bioclimatic zones 1 and 2, respectively (Fig 1B), are the following:

Dfw=exp(−8.82605+1.16201×(lnCd))×F.C. (4)

Dfw=exp(−8.82605+(−1.4682)+(0.33514+1.16201)×lnCd)×F.C. (5)

where: *Dfw* = fiber dry weight (kg), *lnCd* = natural logarithm of crown diameter (cm), exp = exponential, *F*.*C*. = correction factor (1.127).

**Fig 4:**
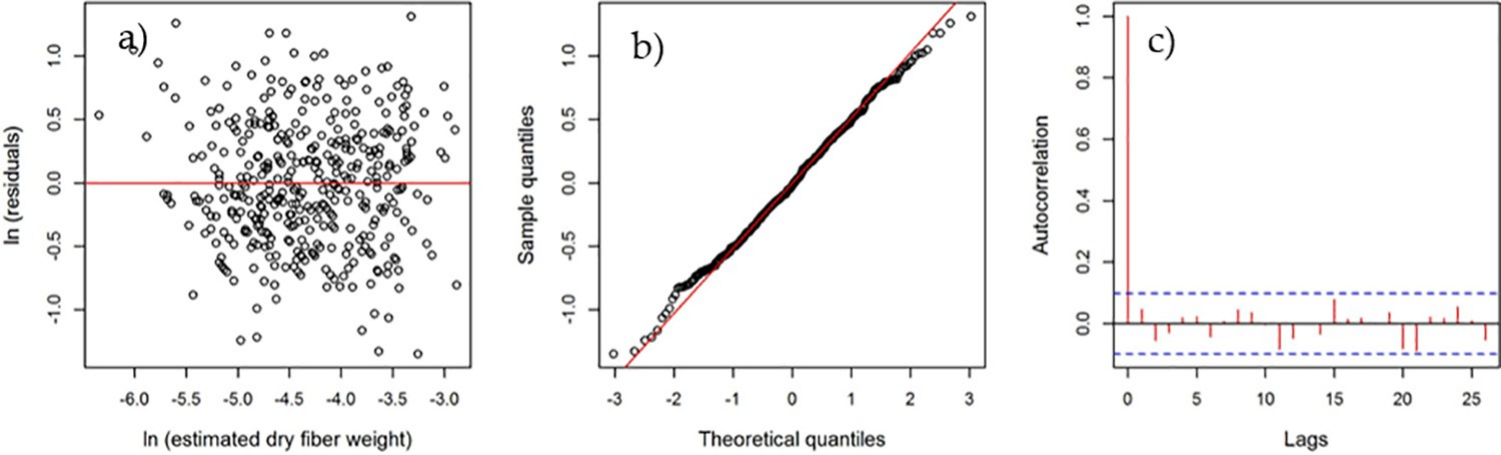
Analysis of residuals of the dummy model for estimating *Agave lechuguilla* Torr. fiber dry weight in northeastern Mexico. Variance homogeneity (a), normality (b), and independence (c).

In accord with the confidence level established (95%), the studentized residuals of the model for predicting *A*. *lechuguilla* dry fiber present homoscedasticity (p = 0.1295) and are normal (p = 0.5343) and independent (p = 0.254) (Fig 4A–4C, respectively), assuring a robust, unbiased and efficient model [15, 28, 29].

### 3.5. Model capacity for predicting dry fiber content of *Agave lechuguilla* Torr.

Model validation using machine learning, with 100 independent data (data that the model would never have “seen”) showed that the model exhibits good capacity for predicting *A*. *lechuguilla* fiber dry weight. The fit statistics (all in logarithmic units) in the four methods of validation (R^2^) are clearly lower (*e*. *g*. average R^2^ of 0.544) than those resulting from the set of training data (R^2^ = 0.629), but very similar to each other (Table 5). This guarantees that, by using the generated equations to predict *A*. *lechuguilla* dry fiber with data that the model has never seen, the estimations are adequate.

**Table 5:** Fit statistics of the evaluation of the predictive capacity of the model for predicting *Agave lechuguilla* Torr. fiber dry weight, using machine learning with an independent sample (n = 100).

Validation	Set data	n	R^2^	RMSE	MAE
**Model fit**	Training	399	0.629	0.487	0.397
**Leave One Out Cross-Validation (LOOCV)**	Test	100	0.512	0.574	0.461
**k-fold validation (Cross-Validation)**	Test	100	0.534	0.564	0.462
**Repeated K-fold (Cross-Validation)**	Test	100	0.545	0.559	0.459
**Bootstrap Validation**	Test	100	0.583	0.561	0.453

n = sample size, R^2^, = coefficient of determination, RMSE = root mean square error, MAE = mean absolute error.

According to the literature, using independent data to validate a model is one of the most recommended techniques [39, 40]. Using a set of independent data and applying the model generated with the training set, Velasco *et al*. [3] evaluated its capacity to predict dry fiber in this species and found that the fit statistics and predictions are very similar, assuring robustness when predicting with new data.

In similar studies conducted in Zone 2 (Fig 1) with n = 287, Blando and Baca [12] found that *Cd* and number of usable leaves explained 76.09% of the *A*. *lechuguilla* fiber. Using 95 individuals (*H* ≥ 25 cm) in Zone 2 of this study, Pando et al. [13] demonstrated that *H* and *Cd* (as a single independent variable) explained 86.9% of *A*. *lechuguilla* fiber dry weight, with an error of 5.041 g. In the same Zone (n = 240) Velasco et al. [3] found that the diameter and height of the bud explained 68% of the fiber in *A*. *lechuguilla*. It is evident that the fit statistics of each model differ from that of our study and from each other due to the sample size (n), sampling method (directed, selective, quadrants), plant selection (minimum height), geographic location of the study (zone 1 or 2), model type (linear, non-linear, simple or multiple), type of predictor and even compliance or non-compliance with the model assumptions.

Although linear models have been generated to estimate fiber weight in *A*. *lechuguilla* [12, 13], the allometry reveals exponential-type changes in dimensions relative to the parts, e. g. in *Agave salmiana* Otto ex. Salm. ssp. crassispina (Trel.) Gentry aboveground biomass follows this pattern [41], as in *Agave salmiana* ssp. Crassispina [42], and as has been demonstrated for prediction of aboveground biomass in *A*. *lechuguilla* [26] and dry fiber in our study, applying the power equation (Eq 1) in its linear form (Eq 2).

### 3.6. Canonical relationship between *A*. *lechuguilla* dry fiber and bioclimatic variables

**Fig 5:**
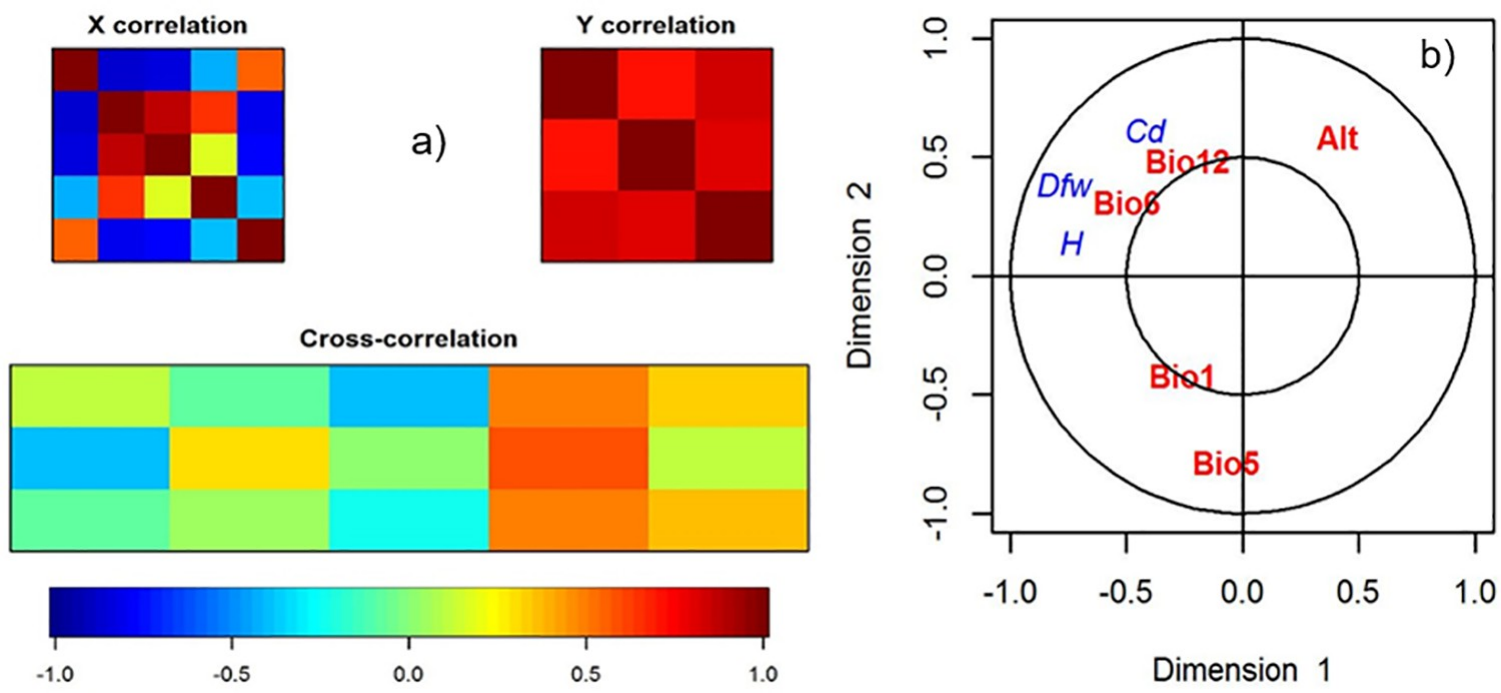
Canonical correlation analysis (CCA) between bioclimatic variables [matrix X: Bio 1 (°C), maximum temperature of the hottest month, Bio 5 (°C), minimum temperature of the coldest month, Bio 6 (°C), and mean annual precipitation, Bio 12 (mm) and altitude (m)]; and Agave lechuguilla Torr. dendrometric variables [matrix Y: crown diameter (Cd, cm), total plant height (H, cm), fiber dry weight (Dfw, kg)] (a) and their expression in the first two dimensions of the CCA.

According to the first and second canonical variable, the degree of correlation between dendrometric variables (matrix *Y*) is higher (r = 0.9000) than that between bioclimatic variables (matrix *X*), which is r = 0.7345 (Fig 5A). The correlation in the first three dimensions is 0.8101, 0.5395 and 0.2510, respectively. Crossed canonical correlation shows that the minimum temperature of the coldest month, Bio 6, and annual precipitation, Bio 12 (Fig 5A, 5B and Table 6), correlated positively with *A*. *lechuguilla* dry fiber production in these bioclimatic zones. According to the three models of general circulation, CCSM4, HadGEM2-AO and MIROC5 [20], in a scenario of moderate CO_2_ emissions (4.5 W/m^2^), it is expected that by 2050, Bio 6 will decrease 0.56°C in the area of *A*. *lechuguilla* distribution, possibly affecting the species’ fiber production, while Bio 12, will decrease only 1.79%.

**Table 6:** Correlation analysis between groups of dendrometric variables of *Agave lechuguilla* Torr. and bioclimatic variables.

	Cd	H	Dfw	Alt	Bio 1	Bio 5	Bio 6	Bio 12
**Cd**	1	0.0006	0.00	0.6703	0.7888	0.0758	0.0292	0.1592
**H**	0.70	1	0.00	0.0940	0.2068	0.9075	0.0075	0.6685
**Dfw**	0.84	0.80	1	0.7177	0.8874	0.2884	0.0258	0.1070
**Alt**	0.10	-0.38	-0.09	1	0.0000	0.0000	0.0557	0.0128
**Bio 1**	-0.06	0.29	0.03	-0.86	1	0.0000	0.0017	0.0000
**Bio 5**	-0.41	0.03	-0.25	-0.84	0.86	1	0.4456	0.0000
**Bio 6**	0.49	0.58	0.50	-0.43	0.66	0.18	1	0.0802
**Bio 12**	0.33	0.10	0.37	0.55	-0.81	-0.76	-0.40	1

*Cd* = crown diameter (cm), *H* = total height (cm), *Alt* = altitude (m), *Dfw* = fiber dry weight (kg), Bio 1 = mean annual temperature (°C), Bio 5 = maximum temperature of the warmest month (°C), Bio 6 = minimum temperature of the coldest month (°C), Bio 12 = annual rainfall (mm).

## 4. Conclusions

The principal components analysis generated two bioclimatic zones, in *Zone* 2 (located in the eastern part of the study area) *A*. *lechuguilla* productivity is higher than in *Zone* 1. This zoning is useful when focusing efforts specifically on management and use of this species. The best predictor of dry fiber content is crown diameter (*Cd*). Using dummy regression, it was possible to establish an equation for each bioclimatic zone (Fig 1B). Validation of this equation revealed high predictive capacity of the model, which is also easy, rapid, and inexpensive to use and is useful in large part of the known distribution area of the species. The results suggest that the species could be vulnerable to climate change with a decrease in Bio 6 of more than 0.5°C by 2050 and there would likely be a gradual reduction in *A*. *lechuguilla* fiber production over the coming years.

## Supporting information

S1 Table *Agave lechuguilla* Torr. individuals evaluated and ejidos sampled by municipality and state. (DOCX)

S2 Table Fit statistics of the power-dummy model in logarithmic form to establish differences in *Agave lechuguilla* Torr. dendrometric variables and bioclimatic variables between zones derived from the PCA. (DOCX)
