# Supplementary material for: Dummy regression to predict dry fiber in Agave lechuguilla Torr. in two large-scale bioclimatic regions in Mexico
Source: PLoS One. 2022 Sep 15;17(9):e0274641. doi: 10.1371/journal.pone.0274641 (PMC9477326; doi:10.1371/journal.pone.0274641)
Supplement: S1 Table — (DOCX) [file pone.0274641.s001.docx]

**S1 Table .** *Agave lechuguilla* Torr. individuals evaluated and ejidos sampled by municipality and state.

| State | Municipality | No. plots | No. Individuals |
| --- | --- | --- | --- |
| Chihuahua | Aldama | 7 | 57 |
|  | Coyame | 7 | 64 |
|  | Jiménez | 1 | 15 |
|  | Ojinaga | 1 | 10 |
| Coahuila | Cuatro Ciénega | 1 | 13 |
|  | General Cepeda | 1 | 26 |
|  | Parras | 3 | 17 |
|  | Ramos Arizpe | 2 | 47 |
|  | San Pedro | 2 | 7 |
|  | Viesca | 1 | 9 |
| San Luis Potosí | Catorce | 2 | 27 |
|  | Cuidad del Maíz | 1 | 17 |
|  | Guadalcázar | 1 | 12 |
|  | Matehuala | 2 | 30 |
|  | Venado | 1 | 15 |
|  | Villa de la Paz | 1 | 11 |
|  | Villa Hidalgo | 1 | 12 |
| Zacatecas | Concepción del Oro | 4 | 51 |
|  | Mazapil | 6 | 44 |
|  | Melchor Ocampo | 1 | 15 |
