# Supplementary material for: Dummy regression to predict dry fiber in Agave lechuguilla Torr. in two large-scale bioclimatic regions in Mexico
Source: PLoS One. 2022 Sep 15;17(9):e0274641. doi: 10.1371/journal.pone.0274641 (PMC9477326; doi:10.1371/journal.pone.0274641)
Supplement: S2 Table — (DOCX) [file pone.0274641.s002.docx]

**S2 Table**. Fit statistics of the power-dummy model in logarithmic form to establish differences in *Agave lechuguilla* Torr. dendrometric variables and bioclimatic variables between zones derived from the PCA.

| Var | Coeff. | Estimate | Std. Error | t-value | Pr(>\|t\|) |
| --- | --- | --- | --- | --- | --- |
| Cd | Intercept ($\beta_{0}$) | 3.736 | 0.033 | 113.057 | < 2e-16 *** |
|  | Zone ($\beta_{1}$) | 0.355 | 0.047 | 7.584 | 2.40e-13*** |
| H | Intercept ($\beta_{0}$) | 3.811 | 0.024 | 160.062 | < 2e-16 *** |
|  | Zone ($\beta_{1}$) | 0.093 | 0.034 | 2.747 | 0.00629** |
| Dfw | Intercept ($\beta_{0}$) | -4.499 | 0.056 | -81.001 | < 2e-16 *** |
|  | Zone ($\beta_{1}$) | 0.330 | 0.079 | 4.192 | 3.41e-05*** |
| Bio1 | Intercept ($\beta_{0}$) | 5.256 | 0.007 | 744.087 | < 2e-16 *** |
|  | Zone ($\beta_{1}$) | -0.001 | 0.010 | -0.055 | 0.956 |
| Bio5 | Intercept ($\beta_{0}$) | 5.794 | 0.007 | 864.090 | < 2e-16 *** |
|  | Zone ($\beta_{1}$) | -0.102 | 0.009 | -10.750 | < 2e-16 *** |
| Bio6 | Intercept ($\beta_{0}$) | 3.633 | 0.032 | 114.852 | < 2e-16 *** |
|  | Zone ($\beta_{1}$) | 0.354 | 0.045 | 7.914 | 2.51e-14*** |
| Bio12 | Intercept ($\beta_{0}$) | 5.832 | 0.017 | 341.712 | < 2e-16 *** |
|  | Zone ($\beta_{1}$) | 0.070 | 0.024 | 2.902 | 0.00391** |
| Alt | Intercept ($\beta_{0}$) | 7.270 | 0.018 | 415.330 | < 2e-16 *** |
|  | Zone ($\beta_{1}$) | 0.040 | 0.025 | 1.620 | 0.106 |
